# Differing clinical characteristics between influenza strains among young healthy adults in the tropics

**DOI:** 10.1186/1471-2334-12-12

**Published:** 2012-01-20

**Authors:** Jonathan Yap, Chi Hsien Tan, Alex R Cook, Jin Phang Loh, Paul A Tambyah, Boon Huan Tan, Vernon J Lee

**Affiliations:** 1 Biodefence Centre, Ministry of Defence, Singapore; 2 Department of Statistics and Applied Probability, National University of Singapore, Singapore; 3 Program in Health Services and Systems Research, Duke-NUS Graduate Medical School, Singapore; 4 Defence Medical and Environmental Research Institute, DSO National Laboratories, Singapore; 5 Department of Medicine, National University Health System, Singapore; 6 Saw Swee Hock School of Public Health, National University of Singapore, Singapore; 7 National Centre for Epidemiology and Population Health, Australian National University, Canberra, Australia

## Abstract

**Background:**

Influenza infections may result in different clinical presentations. This study aims to determine the clinical differences between circulating influenza strains in a young healthy adult population in the tropics.

**Methods:**

A febrile respiratory illness (FRI) (fever ≥ 37.5°C with cough and/or sore throat) surveillance program was started in 4 large military camps in Singapore on May 2009. Personnel with FRI who visited the camp clinics from 11 May 2009 to 25 June 2010 were recruited. Nasal washes and interviewer-administered questionnaires on demographic information and clinical features were obtained from consenting participants. All personnel who tested positive for influenza were included in the study. Overall symptom load was quantified by counting the symptoms or signs, and differences between strains evaluated using linear models.

**Results:**

There were 434 (52.9%) pandemic H1N1-2009, 58 (7.1%) seasonal H3N2, 269 (32.8%) influenza B, and 10 (1.2%) seasonal H1N1 cases. Few seasonal influenza A (H1N1) infections were detected and were therefore excluded from analyses, together with undetermined influenza subtypes (44 (1.5%)), or more than 1 co-infecting subtype (6 (0.2%)). Pandemic H1N1-2009 cases had significantly fewer symptoms or signs (mean 7.2, 95%CI 6.9-7.4, difference 1.6, 95%CI 1.2-2.0, *p *< 0.001) than the other two subtypes (mean 8.7, 95%CI 8.5-9.0). There were no statistical differences between H3N2 and influenza B (*p *= 0.58). Those with nasal congestion, rash, eye symptoms, injected pharynx or fever were more likely to have H3N2; and those with sore throat, fever, injected pharynx or rhinorrhoea were more likely to have influenza B than H1N1-2009.

**Conclusions:**

Influenza cases have different clinical presentations in the young adult population. Pandemic H1N1 influenza cases had fewer and milder clinical symptoms than seasonal influenza. As we only included febrile cases and had no information on the proportion of afebrile infections, further research is needed to confirm whether the relatively milder presentation of pandemic versus seasonal influenza infections applies to all infections or only febrile illnesses.

## Background

Influenza infections arising from different influenza strains may result in different clinical presentations. Determining these different clinical presentations is useful for epidemiological comparison. Furthermore, having knowledge of such clinical symptoms may aid clinicians in determining shifts in influenza strains and managing selected patients at higher risk of developing complications from influenza, especially in settings with poor laboratory resources. Few studies have differentiated the clinical presentation of different influenza strains, particularly in the tropics where the spread of influenza differs from temperate regions [1]. One recent tropical study explored the differences in presentation among various influenza subtypes, but was based on limited number of hospital attendances during the onset of the 2009-H1N1 pandemic [2].

Despite earlier hypotheses that the H1N1-2009 pandemic strain would become the predominant influenza strain in circulation [3], the influenza A (H3N2) and influenza B subtypes that were in circulation before the pandemic continue to circulate globally. This provides the opportunity to compare their clinical features. As older children and young adults were substantially affected by the H1N1-2009 outbreaks [4], this study uses a military respiratory disease surveillance program in tropical Singapore to determine the clinical differences between circulating influenza strains among young healthy adults presenting with febrile respiratory illness [5].

## Methods

This study was carried out as part of a respiratory disease surveillance program within the Singapore military. The febrile respiratory illness (FRI) (fever ≥ 37.5°C with cough and/or sore throat) surveillance program was started in 4 large military camps in May 2009 before community spread of pandemic H1N1-2009 in Singapore [6]. All personnel with FRI who visited the camps' primary healthcare clinics from 11 May 2009 to 25 June 2010 were recruited into the study - this study therefore excludes milder cases, and compares the different clinical symptoms among FRI cases with influenza. Nasal washes from each side of the nose were taken from consenting participants by trained medical staff, placed in viral transport media, and sent to the laboratory for aetiological testing within 24 h. In addition, interviewer-administered questionnaires on demographic information and clinical features of infection were obtained during the clinical consultation, and again at 2 weeks post-consult via telephone interview to identify symptoms present during the entire illness course.

Written informed consent was obtained. Approval was given by the military's Joint Medical Committee for Research, and the respective institutional review boards of the National University of Singapore and Australian National University.

### Laboratory methods

To determine aetiology of the FRI cases, we used the Resplex II (version 2.0, Qiagen, Inc., Valencia, CA, USA) multiplex PCR assay as previously described in other studies [5,7,8]. The Resplex II assay is a multiplex PCR assay coupled with bead array detection technology that is able to detect multiple viruses including influenza A and influenza B [7,8]. For each nasal wash specimen, total nucleic acids were extracted using the DNA minikit (Qiagen, Inc, Valencia, CA, USA) and 5 μl of extract were tested with Resplex II on the LiquiChip 200 Workstation.

Specimens identified as Influenza A positive by the Resplex II assay underwent additional singleplex real-time PCR assays for H1, H3, or pandemic H1N1-2009 as previously described [5,9]. To determine the circulating lineages of influenza, selected specimens were partially sequenced by cDNA synthesis using the uni12primer (AGCAAAAGCAGG) [10] with Transcriptor First Strand cDNA synthesis Kit (Roche Diagnostics, Mannheim, Germany). PCR amplification of the partial H1 gene and H3 gene were carried out with primers described previously [11]. The amplicons were then sequenced using the primers and analysed with DNAstar, Lasergene version 7 (Madison, USA). For influenza B-positive specimens, partial HA gene PCR amplification was obtained with in-house designed primers (HA471F = 5'ACCTCAGGATCTTGCCCTAACG-3' and HA1169R = 5'TGTGTATCCGTGCCAACCTGCAAT-3') and sequenced with the same primers as described for the sequencing of the influenza A strains above.

### Statistical analysis

To fulfil the main aim of the study, we analyzed the overall clinical differences among pandemic influenza A (H1N1-2009), influenza A (H3N2), and influenza B. Few seasonal influenza A (H1N1) infections were detected and these were therefore excluded from analyses, along with influenza cases in which no subtype could be determined due to insufficient sample quantity (44 cases, 1.5%), and patients with multiple co-infecting subtypes (6 cases, 0.2%).

We performed two forms of statistical analysis. The first conditions on influenza subtype and addresses the questions: given a patient is infected by this strain, how will his illness express itself in terms of individual symptoms (or signs), syndromes of pairs of symptoms, and overall symptom load, relative to other aetiologies? Overall symptom load was quantified by counting the symptoms or signs, and differences in overall symptom load between strains evaluated using linear models. Empirical proportions of clinical presentation were used to assess patterns, for each pair of strains, with logistic regression to evaluate differences in symptom expression. We evaluated the presence of paired syndromes by assessing whether the joint distributions of symptom pairs were more or less likely to occur together using binomial tests, defining the excess probability ratio to be the ratio of the proportion of cases in which the pair occurred together relative to the product of the marginal proportion of each symptom.

The second analysis conditions on the suite of symptoms enumerated in this study and asks: given that the patient has expressed this set of symptoms, what is the likely aetiology? In this analysis, potential confounding was addressed by multivariate analyses between pairs of strains, with preliminary models containing all statistically significant variables on univariate analysis across all subtypes, before eliminating non-significant symptom variables sequentially starting with the one with the highest *p*-value. No interactions between variables were significant.

Statistical analyses were performed using R [12], with a significance level of 0.05.

## Results

**Figure 1:**
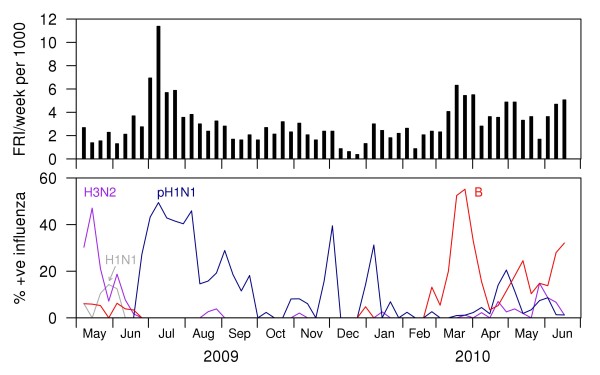
**Distribution of influenza cases during study period**. The top panel presents the weekly number of febrile respiratory infections per 1000 population. The bottom panel presents the proportion positive for each influenza subtype.

A total of 2858 participants (98% of those eligible) were recruited during the study period, with 2717 (95.1%) participants completing the follow-up survey. Participants were mostly young adults (average age 21 years, SD 3) and male (2853, 99.8%) due to the demographics of the military setting. There were a total of 821 influenza cases - of which 434 (52.9%) were pandemic H1N1-2009, 58 (7.1%) seasonal H3N2, 269 (32.8%) influenza B, and 10 (1.2%) seasonal H1N1. The influenza vaccination status of the influenza cases is presented in Table 1. The circulating strains during the study period were the A/California/7/2009(H1N1)-like virus, A/Perth/16/2009-(H3N2)-like virus, and influenza B from the Victoria-lineage, closely related to the B/Brisbane/60/2008-like virus. Figure 1 shows the distribution of influenza cases over the course of the study.

**Table 1:** Influenza vaccination status of influenza cases

Influenza cases	Type of influenza vaccine received			
	
	Nil	Pandemic vaccine	Seasonal vaccine	Both
**H1N1pdm**	345	33	46	10

**H3N2**	40	7	6	5

**H1N1**	10	0	0	0

**B**	41	212	2	14

**Figure 2:**
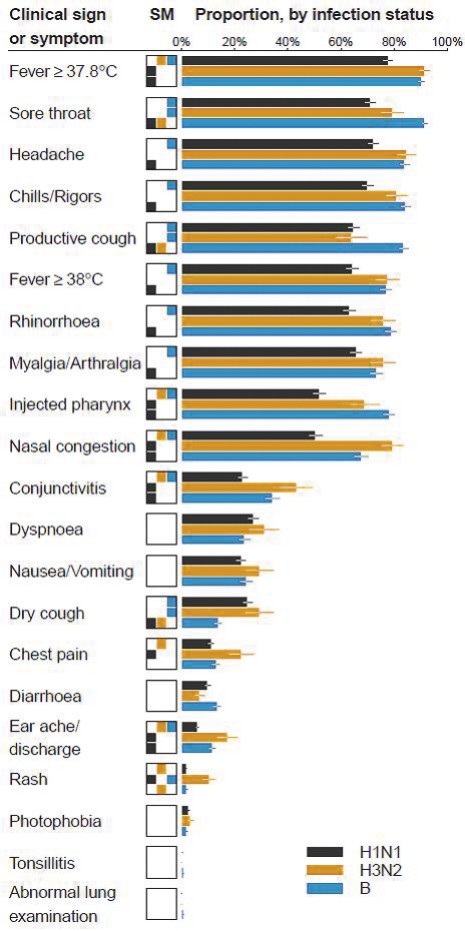
**Univariate comparison of clinical signs or symptoms between influenza subtypes**. Symptoms or signs are ranked by average frequency over the study period. Empirical frequencies of presentation of each symptom or sign are presented in the right column as bars, with 95% confidence intervals represented by whiskers. Symptoms or signs that have statistically discernible differences in frequencies for pairs of strains at the 5% level are indicated in the significance matrix (SM) column. For instance, for a fever of 37.8 or above, pandemic H1N1 is significantly different from the other two strains, indicated by orange and blue cells in the SM in the row for H1N1-2009, and the grey cell in the rows for H3N2 and influenza B, but the latter have no statistically discernible difference, indicated by the white cells in their rows. With 63 tests, the conservative expected number of false discoveries is 3, assuming no differences at all between strains.

From the comparison among the three main influenza strains (pandemic H1N1-2009, seasonal H3N2, and influenza B), pandemic H1N1-2009 cases had significantly fewer symptoms or signs (mean 7.2, 95%CI 6.9-7.4, difference 1.6, 95%CI 1.2-2.0, *p *< 0.001) than the other two subtypes (mean 8.7, 95%CI 8.5-9.0). There were no statistical differences between H3N2 and influenza B (*p *= 0.58). The patterns of symptoms expressed also differed substantially between strains as shown in Figure 2. Pandemic H1N1-2009 cases were significantly less likely to have nasal congestion, eye or ear symptoms, chest pain, headache, high fever, or injected pharynx compared to H3N2; and less likely to have productive cough, sore throat, nasal congestion, rhinorrhoea, eye or ear symptoms, chills or rigors, myalgia or arthralgia, headache, high fever, or injected pharynx, but more likely to have dry cough compared to influenza B. H3N2 cases were associated with more dry cough and rash but less productive cough or sore throat than influenza B.

After adjusting for potential confounders as shown by the multivariate analysis in Table 2, cases with nasal congestion, rash, eye symptoms, injected pharynx or fever were more likely to be infected with H3N2 than pandemic H1N1-2009. Those with sore throat, fever, injected pharynx or rhinorrhoea were more likely to be infected with influenza B than H1N1-2009, while dry cough patients were more likely to be infected with H1N1-2009 than influenza B. Those with nasal congestion or rash were more likely to be infected with H3N2 than influenza B but those with productive cough were more likely to be infected with influenza B than H3N2. None of the subjects required hospitalisation.

**Table 2:** Multivariate analysis comparing clinical features between influenza subtype- positive cases

Influenza H1N1pdm vs H3N2*		
Parameters	Adjusted odds ratio (95% Confidence interval)	*p*value
Blocked nose	0.33 (0.16, 0.72)	0.005

Rash	0.10 (0.02, 0.37)	< 0.001

Eye symptoms	0.47 (0.24, 0.93)	0.03

Injected pharynx	0.45 (0.21, 0.97)	0.04

Fever (≥37.8°C)	0.33 (0.11, 0.99)	0.05

Influenza H1N1pdm vs B*		

**Parameters**	**Adjusted odds ratio (95% Confidence interval)**	***p *value**

Sore throat	0.44 (0.24, 0.80)	0.007

Fever (≥37.8°C)	0.51 (0.29, 0.92)	0.024

Injected pharynx	0.45 (0.29, 0.72)	< 0.001

Age (per year)	1.25 (1.08, 1.46)	0.003

Dry cough	2.10 (1.25, 3.54)	0.005

Running nose	0.54 (0.34, 0.86)	0.009

Influenza H3N2 vs B*		

**Parameters**	**Adjusted odds ratio (95% Confidence interval)**	***p *value**

Blocked nose	3.00 (1.24, 7.19)	0.01

Rash	6.88 (1.50, 31.60)	0.01

Age (per year)	1.28 (1.09, 1.51)	0.002

Cough with sputum	0.24 (1.11, 0.51)	< 0.001

*Age, dry cough, wet cough, sore throat, running nose, blocked nose, sore eyes or eye pain, chills/rigors, myalgia/arthralgia, ear symptoms, rash, chest pain, headache, Fever ≥ 37.8°C, Fever ≥ 38.0°C, and injected pharynx were included in the analysis before non-significant terms were sequentially removed. With 48 tests, the conservative expected number of false discoveries is 2.4.

**Figure 3:**
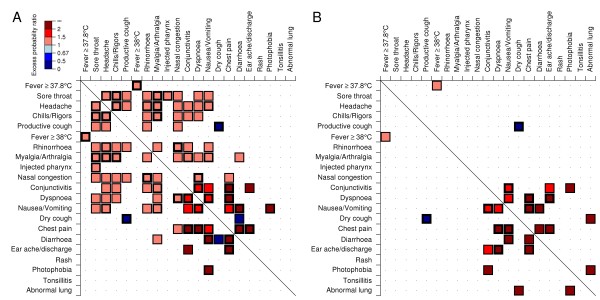
**Symptom or sign complexes for A) H1N1-2009, B) Influenza B**. Symptoms or signs are ranked by average frequency over the study period over all strains. The expected proportion of symptom pairs assuming symptoms develop independently is the product of the two marginal distributions. The discrepancy between this and the observed proportion of symptom pairs is assessed via a binomial test, with differences that are significant at the 5% level represented by coloured cells, with the thickness of the cell wall indicating the *p*-value (thin means 1% <*p *< 5%; medium, 0.1% <*p *< 1%; and thick, *p *< 0.1%). Colours encode the excess probability ratio, a measure of effect size, with blue indicating a lower proportion and red a higher proportion observed than expected, and shade indicating the ratio of the empirical proportion that the pair of symptoms appear together relative to what would be expected if their occurrence were independent. With 420 tests, the conservative expected number of false discoveries is 21, assuming all symptoms or signs occur independently".

Figure 3 shows that some symptoms were significantly associated (or dissociated) with other symptoms. Some are obvious, such as dry and productive coughs which were dissociated, while fever ≥ 37.8°C and fever ≥ 38°C were associated. For pandemic H1N1-2009, some less common symptom pairs appeared together more than twice as often as expected: chest pain was strongly associated with conjunctivitis, dyspnoea, diarrhoea and ear ache, while nausea or vomiting was strongly associated with diarrhoea and photophobia, and conjunctivitis with ear ache. Few patients presented with both diarrhoea and a dry cough. Many of the more common symptoms also appeared as complexes, with the appearance of one increasing the chances of the simultaneous expression of others. For influenza B, chest pain also more frequently occurred if accompanied by dyspnoea, nausea or vomiting, diarrhoea, or ear ache; diarrhoea was often coupled with nausea or vomiting; dyspnoea with ear ache; photophobia with conjunctivitis or abnormal findings on clinical examination of the lungs; while abnormal clinical lung findings more often expressed themselves jointly with a dry cough than expected by chance. There were too few H3N2 cases to identify significant syndromes.

## Discussion

Determining the clinical characteristics between circulating influenza strains is important for epidemiological decision making such as control and surveillance strategies, and for clinicians to understand the symptom complexes for different strains for patient management. Our peri-pandemic period data shows the diversity of clinical presentations among influenza strains - it is important to note that our results may only apply to the specific strains that we studied, and may not apply to other strains in general. Pandemic H1N1-2009 cases presented with fewer symptoms overall compared to seasonal H3N2 or influenza B cases, in spite of the propensity for pandemic H1N1-2009 to affect older children and young adults [4]. This shows that although the extent of spread of the 2009 pandemic was substantial in this population, the clinical severity may have been lower than even seasonal influenza strains, resulting in a lower overall impact compared to previous pandemics. In addition, as influenza spreads quickly in closed environments such as the military, schools, or boarding facilities [13], knowing the clinical characteristics of existing influenza strains might help raise the suspicion of novel influenza strains if marked changes in clinical characteristics are noted, and to identify novel strains for rapid initiation of control measures.

Our study showed that pandemic H1N1 cases were less likely to present with high temperature, sore throat, rhinorrhoea and injected pharynx compared to influenza B cases; and less likely to present with high fever, rash, nasal congestion, eye symptoms and injected pharynx compared to H3N2 cases. A tropical study also found that pandemic H1N1-2009 presented with fewer symptoms than seasonal influenza; including less fever, rhinorrhoea and dyspnoea but more cough [2]. However, yet another found no differences in presentation between pandemic H1N1 and other influenza subtypes [14]. However, the latter two studies did not distinguish between various seasonal influenza subtypes. A study comparing adults with pandemic H1N1-2009 to those with H3N2 found no statistically significant differences for a smaller set of 12 symptoms [15]. In our study, subjects with nasal congestion or rash were more likely to be infected with H3N2 than influenza B but those with productive cough were more likely to be infected with influenza B. Another study from France showed that temperature of > 38.2°C, stiffness or myalgia, rhinorrhoea, and cough were predictive of H3N2 infection, showing that differences exist for different strains in different settings [16].

Although reasons for different symptoms and syndromes caused by influenza subtypes are not immediately apparent, this may be related to host or viral factors which have yet to be elucidated. This should form the basis for further research into the pathogenic properties of influenza subtypes and strains; and the possible linkages to pre-existing immunity or cross-immunity from other strains.

The differences in the clinical symptoms from the influenza strains shown in this study also display the difficulty in using solitary descriptions of influenza (such as traditional ILI definitions) with predictable accuracy for clinical diagnosis and case management. The predictive accuracy for influenza in any setting will depend on the circulating influenza strains and other respiratory pathogens circulating locally. It is therefore important to combine clinical syndromic surveillance with effective laboratory testing. In the context of clinical management, more sophisticated rapid tests may be required to identify influenza cases with high sensitivity and specificity for clinical management and public health measures to be taken.

Our study's strengths are its large sample size, high follow-up rate, and high diagnostic ascertainment of influenza subtypes. Limitations include selection bias towards febrile symptomatic cases - although influenza may present as milder infections, these are difficult to identify through surveillance and we are concerned with more severe clinical presentations resulting in absenteeism. This study involved predominantly young adult males, and results may not be generalizable to the overall population, necessitating further studies to explore differences in presentation among various age groups and gender. Finally, the actual clinical impact of differentiating between various influenza subtypes may be limited and would depend on factors such as their current anti-viral resistance profiles. However, this is not examined in this study, and strains displaying anti-viral resistance may have different clinical presentations.

## Conclusion

Different influenza strains have different clinical presentations in the young adult population, and pandemic H1N1 influenza cases had fewer and milder clinical symptoms than seasonal influenza H3N2 and B.

## Competing interests

VJL has received unrelated research support from GSK. PAT has received research support and honoraria from Baxter, Adamas, Merlion Pharma, and Novartis as well as travel support from Pfizer and Wyeth and sits on the boards of the Asia Pacific Advisory Committee on Influenza and the Asian Hygiene Council. The rest of the authors declare that we do not have any conflict of interests, financial or otherwise, in this study.

## Authors' contributions

JY and VJL conceived the study, collected the data, performed the analysis, and wrote the manuscript together. CHT, ARC, JPL and BHT performed the analysis and participated in the manuscript writing. PAT participated in the manuscript writing. All authors have read and approved the final manuscript and the manuscript is currently not submitted for publication elsewhere.

## Funding source

The work was supported by a Singapore Ministry of Defence funded operational research program. The funders had no role in study design, data collection and analysis, decision to publish, or preparation of the manuscript.

## Pre-publication history

The pre-publication history for this paper can be accessed here:

http://www.biomedcentral.com/1471-2334/12/12/prepub
